# Shin'iseihaito (Xinyiqingfeitang) Suppresses the Biofilm Formation of* Streptococcus pneumoniae* In Vitro

**DOI:** 10.1155/2017/4575709

**Published:** 2017-04-16

**Authors:** Masaaki Minami, Toru Konishi, Hiroshi Takase, Toshiaki Makino

**Affiliations:** ^1^Department of Bacteriology, Graduate School of Medical Sciences, Nagoya City University, 1 Kawasumi, Mizuho-ku, Nagoya, Japan; ^2^Department of Pharmacognosy, Graduate School of Pharmaceutical Sciences, Nagoya City University, 3-1 Tanabe-Dori, Mizuho-ku, Nagoya, Japan; ^3^Core Laboratory, Graduate School of Medical Sciences, Nagoya City University, Nagoya, Japan

## Abstract

*Streptococcus pneumoniae* (*S. pneumoniae*) is the important pathogen that causes otolaryngeal diseases such as sinusitis.* S. pneumoniae* frequently forms the biofilm to prevent severe circumstances such as antimicrobial agents. Shin'iseihaito (xinyiqingfeitang) is a formula of Japanese traditional Kampo medicine that has 9 crude drugs and provides the medicinal usage for sinusitis. The objective of the present study is to reveal the mechanism of antibiofilm activity by Shin'iseihaito extract (SSHT). SSHT significantly inhibited the formation of biofilm from* S. pneumoniae* ATCC 49619 in dose- and time-dependent manners. SSHT also significantly suppressed the biofilm formation by other five different cps types of* S. pneumoniae* clinical isolates. We found that the extracts of 8 out of 9 components in Shin'iseihaito had the inhibitory effects of biofilm formation, and the extract of the root of* Scutellaria baicalensis* had the strongest effect among the ingredients of Shin'iseihaito. We found that the capsule of SSHT-treated* S. pneumoniae* was significantly thinner than that of the untreated group and that SSHT reduced the hydrophobicity of bacterial cell surface. Our results suggest that Shin'iseihaito may be a useful agent for the treatment of* S. pneumoniae*-induced sinusitis because of the inhibition of biofilm formation of* S. pneumoniae*.

## 1. Introduction

*Streptococcus pneumoniae* (*S. pneumoniae*) causes sinusitis, otitis media, pneumonia, meningitis, and sepsis in young children and the elderly particularly [1]. It colonizes the human nasopharynx during the first months of life, where it can persist as part of the commensal flora. Streptococcal colonization and its disease are often associated with biofilm formation [2]. The presence of biofilm in human biopsy specimens of the sinus and middle ear mucosa has been reported [3, 4].

Biofilms are biotic and abiotic surfaces associated with highly structured sessile complex and entrench bacterial communities themselves in a self-produced extracellular matrix of exopolysaccharide (EPS) along with proteins and micromolecules such as DNA [5]. Bacteria that adhere to implicated medical devices or damaged tissue can cause persistent infections through biofilm formation [6]. Conventionally, antibiotics used to treat these biofilm-forming pathogens are not targeting the biofilms; rather they target their planktonic counterparts, which means that the antibiotic creates the pressure on the bacteria and the bacteria get resistance against the drug [7, 8]. Although the treatments against biofilm-associated bacteria are not currently established, the novel drug which have antibiofilm formation will be desired to overcome the difficult-to-treat infections.

In previous studies, some agents are reported to have the inhibitory effect on biofilm formation. Among Japanese Kampo formula, the extract of rokumigan that is composed of 6 crude drugs prevented the biofilm formation of* Fusobacterium nucleatum* [9]. Amidase LytA inhibited the growth of biofilm of* S. pneumoniae* [10]. S-carboxymethylcysteine inhibited the bacterial adhesion of the human alveolar epithelial cells [11]. Neuraminidase inhibitors such as zanamivir and oseltamivir inhibited the capacity of the bacteria to form sialic acid-dependent biofilms. Sinefungin, S-adenosyl-L-methionine analogue, also inhibited in vitro biofilm growth and in vivo middle ear colonization of* S. pneumoniae* [12]. The inhibitory effect of N-acetyl-L-cysteine (NAC), xylitol, and carrageenan on* S. pneumoniae* biofilm formation in vitro has been reported [13]. However, the information of the effect of these agents is limited, and further investigation about antibiofilm drug will be needed.

Shin'iseihaito (xinyiqingfeitang) is a formula of traditional Japanese Kampo medicine and traditional Chinese medicine both of which are originated from ancient Chinese medicine, which is used for the treatment of upper respiratory tract disease, especially sinusitis [14, 15]. In our previous studies, we investigated the antimicrobial effect of Shin'iseihaito extract (SSHT) in a pneumococcus-infected model [16], the antibacterial activity of SSHT extract against* S. pneumoniae* in vitro [17], and the preventive effect of SSHT in an ovalbumin-induced allergic rhinitis model [18]. However, experimental evidences on the use of SSHT for the treatment of bacterial sinusitis are still limited. Furthermore, the antibiofilm activity of SSHT against* S. pneumoniae* has been unclear yet.

The current study was focused on investigating the antibiofilm effect of SSHT on biofilm formation of* S. pneumoniae* for choosing this formula as antibiofilm drug.

## 2. Materials and Methods

### 2.1. Bacteria

*Streptococcus pneumoniae* (*S. pneumoniae*) ATCC 49619 (cps19F) and five clinical isolates (NCU1, NCU3, NCU5, NCU9, and NCU12) from Nagoya City University hospitals were used in this study. The cps types of these* S. pneumoniae* were as follows: NCU1 cps4; NCU3 cps14; NCU5 cps6ABC; NCU9 cps 9; NCU12 cps 19A.* S. pneumoniae* was usually precultured in Trypticase soy agar with 5% sheep blood (Becton Dickinson, NJ, USA) for 1 day at 37°C under 5% CO_2_ atmosphere.

### 2.2. Crude Drugs

Shin'iseihaito (xinyiqingfeitang) (for daily human dose) consists of 1.5 g of the rhizome of* Anemarrhena asphodeloides* (AA), 0.75 g of the rhizome of* Cimicifuga heracleifolia* (CH), 0.5 g of the leaf of* Eriobotrya japonica *(EJ), 3.0 g of* Gypsum fibrosum *(GF), 0.75 g of the fruit of* Gardenia jasminoides *(GJ), 1.5 g of the bulb of* Lilium lancifolium *(LL), 1.5 g of the flower of* Magnolia salicifolia *(MS), 3.0 g of the tuber of* Ophiopogon japonicus *(OJ), and 1.5 g of the root of* Scutellaria baicalensis *(SB). The mixture of these crude drugs that were standardized by Japanese Pharmacopoeia 17th Edition [19] was boiled in water and filtered. The decoction was dried to yield a powdered extract (SSHT, 2.5 g for daily human dose). SSHT (Lot: 14B019) was provided as a generous gift from the Kobayashi Pharmaceutical (Osaka, Japan). SSHT was suspended in distilled water to prepare the stock solution at a concentration of 0.1 g/mL and kept in −20°C until use. Each 5 g of crude drug described above that was purchased from Daiko Shoyaku (Nagoya, Japan) or Tsumura (Tokyo, Japan) was boiled in 20-time weight of water for 30 min and filtered. Each decoction was lyophilized, and the dried powdered extracts of each crude drug were stored in desiccated condition until use.

### 2.3. Biofilm Assay

Overnight cultures of* S. pneumoniae* strains ATCC 49619 (10^6^ CFU) were seeded into 96-well polystyrene plates (Thermo Fisher Scientific, MA, USA), which were incubated with Todd Hewitt broth (Becton Dickinson) with 0.3% yeast extract (Becton Dickinson) (THY) medium with or without SSHT (500 *µ*g/mL) at 37°C for 1, 2, or 3 days. After the removal of medium, the plates were washed three times with PBS, and then the adherent bacteria were stained with 0.2% crystal violet at room temperature for 10 min and gently washed three times with phosphate buffered saline (PBS). Each biomass was quantitated by measuring absorbance at 570 nm (A570). Wells incubated without bacteria were used as blanks. The absorbance for the blank wells was subtracted from the test values. As dose-dependent analysis, we also used three kinds of concentrations of SSHT (5, 50, and 500 *µ*g/mL). For confocal microscopic observations, the bacteria were grown on glass coverslips placed in 24-well polystyrene plates (Thermo Fisher Scientific) at 37°C for 2 days. After the removal of the medium, the wells were washed three times with PBS and stained with fluorescein isothiocyanate isomer (FITC) (Wako Pure Chemical, Osaka, Japan) according to the manufacturer's instructions; then images were taken with an LSM 510 confocal laser microscope (Carl Zeiss, Oberkochen, Germany). Three-dimensional images were created from Z-stack images using Imaris software (Carl Zeiss). The thickness of each biofilm was also measured using Imaris software.

### 2.4. Morphologic Investigation of Bacteria

To study the bacteria morphologically using transmission electron microscopy (TEM) JEM1011J (JEOL, Tokyo, Japan), the* S. pneumoniae* strains ATCC 49619 (10^6^ CFU) treated with or without SSHT (500 *µ*g/mL) were cultured in THY medium for 1 day as a first step. For negative staining, approximately one drop of the bacterial culture was applied onto a 300-mesh carbon formvar copper grid (Nisshin EM, Tokyo, Japan). Then, the excess of solution was removed and negative staining was done by 2% phosphotungstic acid (PTA) (Wako Pure Chemical). Then, the samples were observed by electron microscopy. Digital images were taken with a MegaView Slow-scan camera (JEOL).

### 2.5. Analysis of Cell Surface Hydrophobicity

The hydrophobicity of cell surfaces was determined using the hexadecane method with a minor modification [20]. Briefly,* S. pneumoniae* ATCC 49619 strain treated with or without SSHT (500 *µ*g/mL) was grown to the exponential phase and suspended in PBS whose absorbance at 600 nm (A600) was adjusted to 1.0. After the addition of 200 *μ*L of n-hexadecane to 2 mL of bacterial suspensions in glass tubes, the A600 value of the lower aqueous phase was measured. Then, the tubes were vigorously vortexed for 2 min, followed by 10 min of incubation at room temperature to allow for phase separation, and the A600 value of the lower aqueous phase was measured. The hydrophobicity was calculated using the following equation: percent of hydrophobicity = [1 − (A600 after vortexing/A600 before vortexing)] × 100.

### 2.6. Statistical Analysis

Experimental data were expressed as mean values with standard deviation (SD). Statistical analysis of the differences between the mean values obtained was performed using Tukey's multiple comparison tests for multiple groups and an unpaired Student's or Weltch's *t*-test for two groups, and the statistical difference was considered with* p* < 0.05.

## 3. Results

### 3.1. Microplate Analysis

**Figure 1:**
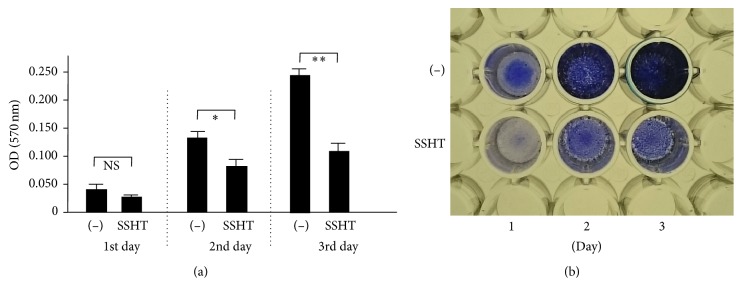
SSHT inhibits the biofilm formation of* S. pneumoniae *in time-dependent manners. (a)* S. pneumoniae* was treated with or without SSHT (500 *µ*g/mL) for 1–3 days, and its antibiofilm activity was quantified by crystal violet adsorption by measuring absorbance at 570 nm. Data shown represent the mean ± SD (*n* = 6). ^*∗*^*p* < 0.05; ^*∗∗*^*p* < 0.01. NS: not significant. (b) Image of microplate. SSHT: Shin'iseihaito extract.

**Figure 2:**
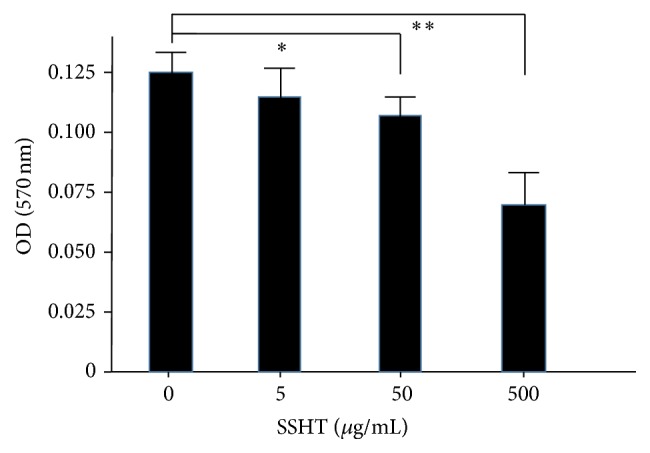
SSHT inhibits the biofilm formation of* S. pneumoniae *in dose-dependent manners.* S. pneumoniae* was treated with SSHT (0, 5, 50, and 500 *µ*g/mL) for 2 days, and its antibiofilm activity was quantified by crystal violet adsorption by measuring absorbance at 570 nm. Data shown represent the mean ± SD (*n* = 6). ^*∗*^*p* < 0.05; ^*∗∗*^*p* < 0.01. SSHT: Shin'iseihaito extract.

**Figure 3:**
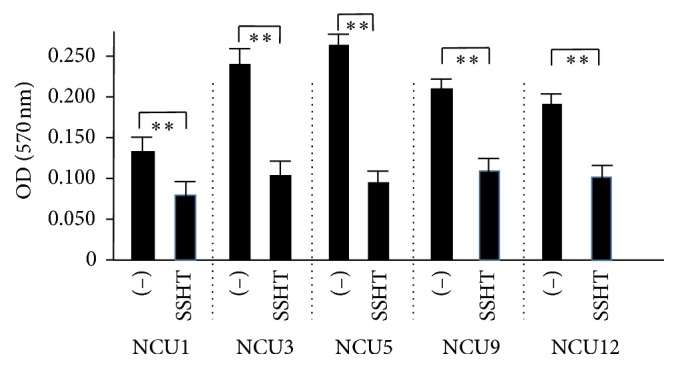
SSHT inhibits the biofilm formation of other five cps types of* S. pneumoniae. *Five different strains of* S. pneumoniae* were treated with or without SSHT (500 *µ*g/mL) for 2 days, and its antibiofilm activity was quantified by crystal violet adsorption by measuring the absorbance at 570 nm. Data shown represent the mean ± SD (*n* = 6). ^*∗∗*^*p* < 0.01. The cps types of these* S. pneumoniae *were as follows: NCU1 cps4; NCU3 cps14; NCU5 cps6ABC; NCU9 cps 9; NCU12 cps 19A. SSHT: Shin'iseihaito extract.

To evaluate whether SSHT could inhibit the biofilm formation or not,* S. pneumoniae* was grown in Todd Hewitt broth with 0.3% yeast extract (THY) medium with or without SSHT, and the ability to form biofilm on polystyrene plates was assessed by crystal violet staining. As expected, SSHT significantly inhibited the formation of biofilm from* S. pneumoniae* ATCC 49619. Although there is no statistical difference in day 1, the significant inhibitory effect of SSHT on* S. pneumoniae *biofilm formation was found in day 2 (*p *< 0.05) and day 3 (*p* < 0.01) (Figure 1). In day 2, SSHT (5 *μ*g/mL) did not inhibit the biofilm formation; however, 50 *μ*g/mL (*p* < 0.05) and 500 *μ*g/mL (*p* < 0.01) of SSHT significantly inhibited the biofilm formation of* S. pneumoniae*, respectively (Figure 2). Thus, we confirmed that antibiofilm activity of SSHT was in dose- and time-dependent manners. To rule out the specific phenomenon of one* S. pneumoniae* (ATCC 49619), we investigated the antibiofilm effect of SSHT against other five* S. pneumoniae* clinical isolates after 2 days' incubation.  Figure 3 showed that SSHT also significantly suppressed the biofilm formation of the other five* S. pneumoniae* clinical isolates (*p* < 0.01).

### 3.2. Confocal Laser Scanning Microscopy

**Figure 4:**
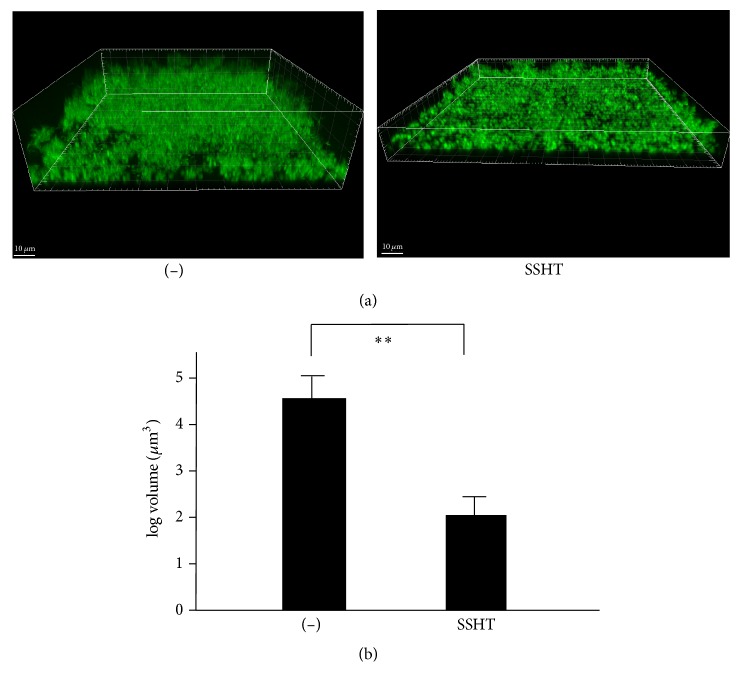
Effect of SSHT on the thickness of* S. pneumonia* biofilm. (a) 3D analysis of biofilm formation of* S. pneumonia *treated with SSHT. Bacteria were cultured with or without SSHT (500 *µ*g/mL) under static conditions at 37°C for 2 days in THY. Formed biofilms were stained with FITC. Three-dimensional images were reconstructed from confocal optical sections using Imaris software. Thickness was measured at nine arbitrary points in each field using Imaris software. (b) The quantification of the biofilm of* S. pneumonia *treated with SSHT. The bacteria were cultured with or without SSHT (500 *µ*g/mL) under static conditions at 37°C for 2 days in THY, and the thickness was measured at nine arbitrary points in each field using Imaris software. Data shown represent the mean ± SD (*n* = 6). ^*∗∗*^*p* < 0.01. SSHT: Shin'iseihaito extract.

To confirm the data obtained by crystal violet staining, the biofilms formed by* S. pneumoniae* ATCC 49619 treated with or without SSHT after 2 days were stained with FITC dye and viewed using confocal laser scanning microscopy (Figure 4(a)). The obtained Z-stack images were converted into three-dimensional images; then, the thickness of the biofilms was measured. The biofilms formed by the control showed a multilayered surface-adhered cluster reflecting a mature biofilm with an average thickness of 80 *μ*m. In contrast, the SSHT-treated strains showed a decreased biofilm formation, with an average thickness of 10 *μ*m. The quantification analysis of biofilm volume showed that the biofilm-volume levels in microbe treated with SSHT were significantly lower than those untreated (*p *< 0.01) (Figure 4(b)). Overall, the findings from crystal violet staining were supported by the results from the microscopic three-dimensional observations.

### 3.3. Effect of Each Extract of Shin'iseihaito Component

**Figure 5:**
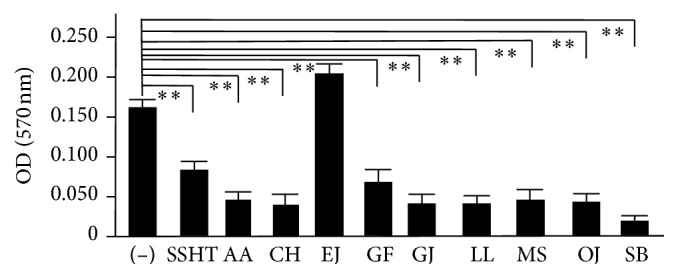
The inhibitory effects of the extracts of each component of Shin'iseihaito on the biofilm formation of* S. pneumoniae. S. pneumoniae* was treated with or without each extract of the components of Shin'iseihaito and SSHT (500 *µ*g/mL) for 2 days, and its anti-antibiofilm activity was quantified by crystal violet adsorption by measuring the absorbance at 570 nm. Data shown represent the mean ± SD (*n* = 6). ^*∗∗*^*p* < 0.01. SSHT: Shin'iseihaito extract; AA: the rhizome of* Anemarrhena asphodeloides*; CH: the rhizome of* Cimicifuga heracleifolia*; EJ: the leaf of* Eriobotrya japonica*; GF:* Gypsum fibrosum*; GJ: the fruit of* Gardenia jasminoides*; LL: the bulb of* Lilium lancifolium*; MS: the flower of* Magnolia salicifolia*; OJ: the tuber of* Ophiopogon japonicus*; SB: the root of* Scutellaria baicalensis.*

We evaluated which components of Shin'iseihaito had antibiofilm activity by crystal violet staining analysis. We prepared the extracts of each component of Shin'iseihaito, and the inhibitory effects of them are shown in Figure 5. Eight kinds of extracts prepared from each component of Shin'iseihaito except for the leaf of* Eriobotrya japonica* significantly inhibited the biofilm formation of* S. pneumoniae*, respectively (*p *< 0.01). Among them, the extract of the root of* Scutellaria baicalensis* had the strongest activity of antibiofilm formation (*p *< 0.01).

### 3.4. The Expression of Capsule of* S. pneumoniae*

**Figure 6:**
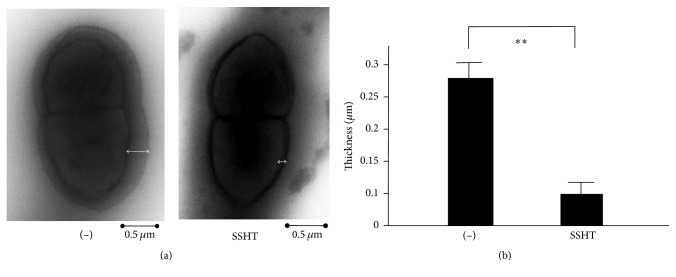
Morphological change of* S. pneumonia *treated with SSHT. (a) Representative photo of* S. pneumoniae* treated with or without SSHT (500 *µ*g/mL) for 1 day using electron microscopy by negative staining. Double-headed white arrow represent the size of bacterial capsule. (b) The change of capsule thickness of* S. pneumonia *treated with SSHT.* S. pneumoniae* was treated with or without SSHT (500 *µ*g/mL) for 1 day, and the thickness was measured at six arbitrary points in the bacteria treated with or without SSHT. Data shown represent the mean ± SD (*n* = 6). ^*∗∗*^*p* < 0.01. SSHT: Shin'iseihaito extract.

We tried to assess the direct effect of SSHT against* S. pneumoniae* ATCC 49619 by morphological analysis. Negative staining analysis revealed that there was no significant difference in the size of the bacteria between SSHT-treated and untreated groups. However, the capsule of* S. pneumoniae* treated with SSHT was thinner than that of* S. pneumoniae* untreated (*p *< 0.01) (Figure 6).

### 3.5. The Hydrophobicity of the Bacterial Cell Surface

**Figure 7:**
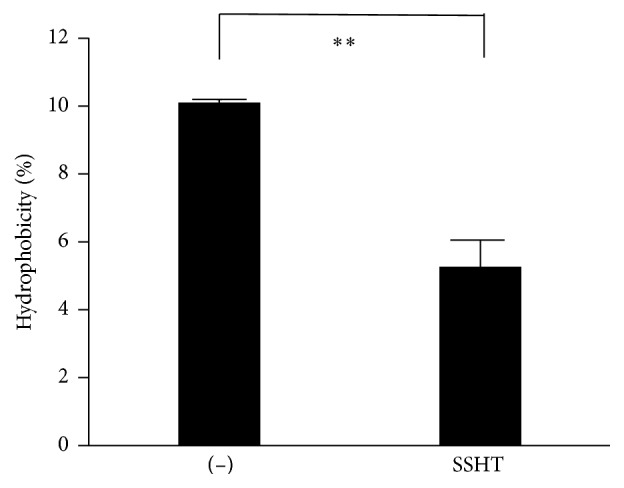
Hydrophobicity of* S. pneumonia *treated with SSHT.* S. pneumoniae* was treated with or without SSHT (500 *µ*g/mL) at exponential phase and the cell surface hydrophobicity grown to the exponential phase (A600 of 1.0) was determined. The value of the control strain was set at 100%. Data shown represent the mean ± SD (*n* = 6). ^*∗∗*^*p* < 0.01. SSHT: Shin'iseihaito extract.

Since the biofilm formation is often associated with the hydrophobicity of the bacterial cell surface, we determined the hydrophobicity of the strains using an n-hexadecane method (Figure 7). The surface hydrophobicity was markedly reduced in* S. pneumoniae* treated with SSHT compared to that of the untreated group (*p *< 0.01).

## 4. Discussion

To our knowledge, this is the first study on the antibiofilm effect of Shin'iseihaito, which is one of the formulas used in Japanese traditional Kampo and traditional Chinese medicine. We clarified the effect of SSHT by microplate, confocal laser microscopy, and transelectronic microscopy analysis.

First of all, our study demonstrated that SSHT significantly inhibited the formation of biofilm from* S. pneumoniae *ATCC 49619. In addition, anti-*S. pneumoniae* biofilm activity of SSHT extract was in dose- and time-dependent manners. We found no differences of biofilm formation between SSHT-treated and untreated* S. pneumoniae* after 1 day, since the biofilm formation might be immature because of good nutritional condition around the bacteria. In general, a poor environment around the bacteria promotes the biofilm formation [13].

Next, SSHT also significantly suppressed the biofilm formation in other five different cps types of* S. pneumoniae* clinical isolates. Although we only investigated different six cps types of* S. pneumoniae*, we supposed that SSHT had antibiofilm activity against* S. pneumoniae* regardless of cps types. This result suggests that SSHT treatment would be more beneficial than pneumococcal vaccine therapy, because different cps types of pneumococcal vaccines are ineffective.

Three-dimensional analysis by confocal laser microscopy confirmed that the biofilm-volume levels of microbes treated with SSHT were significantly lower than those of the untreated microbes. In the electron microscopy methods such as scanning electron microscopy, it is necessary to dehydrate the sample, whereas in the confocal laser fluorescence microscope, it is possible to study the three-dimensional structure of the untreated biofilm.

We found that the extract of 8 out of 9 components of Shin'iseihaito had antibiofilm activity, and the extract of the root of* Scutellaria baicalensis* had the strongest inhibitory effect of biofilm formation among nine components in Shin'iseihaito. When* Burkholderia cenocepacia* biofilms were treated with quorum sensing inhibitors (QSI) such as baicalin hydrate which is one of the ingredients of the root of* Scutellaria baicalensis*,* B. cenocepacia* are impaired in their ability to maintain cells within the biofilm [21]. When used alone, baicalin hydrate resulted in a minor reduction in the number of* B. multivorans* and* B. cenocepacia* cells [21]. Treatment with tobramycin in combination with baicalin hydrate on* B. cenocepacia* and* B. multivorans* biofilms for 24 hours resulted in significantly more killing activity relative to the treatment with tobramycin alone [21]. Another report showed that baicalin had potent inhibitory effects on the adhesion of the nonalbicans* Candida* cells and displayed substantial inhibitory effects on nonalbicans* Candida* biofilm [22]. From these results, it is considered that the main active component of Shin'iseihaito is the root of* Scutellaria baicalensis*, and one of the active compounds is baicalin. Thus, SSHT may suppress the* S. pneumoniae* biofilm formation by regulation of quorum sensing.

We also found that the capsule of SSHT-treated* S. pneumoniae* was thinner than that of the untreated group by negative staining analysis. For the morphological analysis of the bacteria, embedding fixation assay is more popular than negative staining by using transmission electron microscopy. However, the embedding fixation assay may destroy the structure of bacterial surface, especially capsule. The negative staining is the best assay to investigate the bacterial capsule because an extra procedure such as embedding fixation is not needed. Thus, we chose negative staining assay by using transmission electron microscopy.

Furthermore, in order to explain the reduction of capsule under SSHT treatment, we hypothesized that SSHT affected the capsule of* S. pneumoniae* directly. Among the natural products, saponins may be considered as the candidate of direct effect against bacteria. Among the components of Shin'iseihaito, the bulb of* Lilium lancifolium*, the rhizome of* Anemarrhena asphodeloides*, and the tuber of* Ophiopogon japonicas* contain triterpenoid or steroid saponins [23–25]. The amphipathic nature of the saponins indicates their activity as surfactants that can be used to enhance the penetration of macromolecules such as proteins through cell membranes [26]. Saponins are the diverse group of compounds widely distributed in plant world and are characterized by their structure containing a triterpene or steroid aglycone and one or more sugar chains [27]. As commercially significant products, saponins have expanding applications in food, cosmetics, and pharmaceutical industries due to their anticancer, antioxidant, antihypertensive, and antimicrobial activities [23]. On account of their antibacterial and antifungal effects, saponins presumably serve the plants for defence against infections. The fact that saponins can enhance the susceptibility of some bacteria against certain antibiotics in vitro and in vivo was observed in many studies. A higher uptake of the antibiotic drugs into the bacterial cell might have been induced by the interaction of the saponins with the bacterial membrane and, hence, have been responsible for the observed beneficial effects [28]. Saponin-rich extracts from guar meal and* Quillaja* exhibited antibacterial activity against* Staphylococcus aureus *[29]. Saponins from the leaves and barks of* Acacia arabica *have antibacterial activity against diarrheagenic* Escherichia coli* [30]. Korean red ginseng saponins may exert antifungal activity by disrupting the structure of cell membrane [31]. Quorum sensing, related to cell-to-cell signalling, plays a role in cell attachment and detachment from the biofilm [32]. Although the exact role of quorum sensing in various stages of biofilm formation, maturation, and dispersal and in biofilm resistance is not entirely clear, the use of quorum sensing inhibitors (QSI) has been proposed as a potential antibiofilm strategy [33]. A previous report suggested that saponins may make resistant bacteria resensitize to the antibiotics by inhibiting quorum sensing [34]. The three components of Shin'iseihaito, the bulb of* Lilium lancifolium*, the rhizome of* Anemarrhena asphodeloides*, and the tuber of* Ophiopogon japonicas,* may be associated with the inhibition of quorum sensing. Our results also showed that the leaf of* Eriobotrya japonica* did not exhibit antibiofilm activity, since this crude drug may contain a little amount of saponins. Further investigation about the relationship between the content of saponins and their antibiofilm effect in each crude drug will be needed.

The hydrophobic properties of microbial cell surface play crucial roles in bacterium-host cell interactions [33]. Previous investigation showed the interference of plant extracts on the hydrophobicity of Gram-negative bacteria [34] and Gram-positive bacteria [35], thus inhibiting biofilm formation. Similarly, SSHT was able to decrease the hydrophobicity of the cell surface properties of* S. pneumoniae* isolates about twofold and thereby significantly contributed to antibiofilm activity. Bacteria inside biofilms can produce periplasmic glucans which bind to antibiotics, sequestering them in the periplasm and preventing them from the action of antibiotics [36]. Thus, reducing the hydrophobicity will expose the bacterial biofilm to antibiotics and sequentially will ease the eradication of the biofilm.

In conclusion, this is the first study focused on antibiofilm activity of Shin'iseihaito. Therefore, Shin'iseihaito can be used as lead formula for the development of novel antibiofilm agents against* S. pneumoniae*.
